# National Scale Real-Time Surveillance of SARS-CoV-2 Variants Dynamics by Wastewater Monitoring in Israel

**DOI:** 10.3390/v14061229

**Published:** 2022-06-06

**Authors:** Itay Bar-Or, Victoria Indenbaum, Merav Weil, Michal Elul, Nofar Levi, Irina Aguvaev, Zvi Cohen, Virginia Levy, Roberto Azar, Batya Mannasse, Rachel Shirazi, Efrat Bucris, Orna Mor, Alin Sela Brown, Danit Sofer, Neta S. Zuckerman, Ella Mendelson, Oran Erster

**Affiliations:** 1 Central Virology Laboratory, Israel Ministry of Health, Chaim Sheba Medical Center, Ramat Gan 5262000, Israel; itay.baror@sheba.health.gov.il (I.B.-O.); viki.indenbaum@sheba.health.gov.il (V.I.); merav.weil@sheba.health.gov.il (M.W.); michal.elul@sheba.health.gov.il (M.E.); nofar.levi@sheba.health.gov.il (N.L.); irina.aguvaev@sheba.health.gov.il (I.A.); zvi.cohen2@sheba.health.gov.il (Z.C.); virgini.levy@sheba.health.gov.il (V.L.); roberto.azar@sheba.health.gov.il (R.A.); batya.mannasse@sheba.health.gov.il (B.M.); rachel.shirazi@sheba.health.gov.il (R.S.); efrat.bucris@sheba.health.gov.il (E.B.); orna.mor@sheba.health.gov.il (O.M.); alinselabrown@gmail.com (A.S.B.); danit.sofer@sheba.health.gov.il (D.S.); neta.zuckerman@sheba.health.gov.il (N.S.Z.); ella.mendelson@sheba.health.gov.il (E.M.); 2 Sackler Faculty of Medicine, School of Public Health, Tel-Aviv University, Tel-Aviv 69978, Israel

**Keywords:** SARS-CoV-2, variant B.1.1.7 (Alpha), RT-qPCR, wastewater surveillance, differential PCR

## Abstract

In this report, we describe a national-scale monitoring of the SARS-CoV-2 (SC-2) variant dynamics in Israel, using multiple-time sampling of 13 wastewater treatment plants. We used a combination of inclusive and selective quantitative PCR assays that specifically identify variants A19/A20 or B.1.1.7 and tested each sample for the presence and relative viral RNA load of each variant. We show that between December 2020 and March 2021, a complete shift in the SC-2 variant circulation was observed, where the B.1.1.7 replaced the A19 in all examined test points. We further show that the normalized viral load (NVL) values and the average new cases per week reached a peak in January 2021 and then decreased gradually in almost all test points, in parallel with the progression of the national vaccination campaign, during February–March 2021. This study demonstrates the importance of monitoring SC-2 variant by using a combination of inclusive and selective PCR tests on a national scale through wastewater sampling, which is far more amendable for high-throughput monitoring compared with sequencing. This approach may be useful for real-time dynamics surveillance of current and future variants, such as the Omicron (BA.1, BA.2) and other variants.

## 1. Introduction

Since its emergence in December 2019, SARS -CoV-2 (SC-2) spread worldwide, causing the COVID-19 pandemic, with unprecedented impact on human lives, economy, social aspects, and public health throughout the world [1]. While SC-2 infection may cause a range of respiratory, gastrointestinal, and systemic symptoms, the majority of individuals show little to no symptoms [2,3,4,5,6,7] Clinical testing of individuals for SC-2 is the primary surveillance method for obtaining information for implementation of public health strategic interventions, such as quarantine, to mitigate the spread of the virus. The current gold standard for clinical testing is reverse transcriptase quantitative polymerase chain reaction (RT-qPCR) [3], which detects the viral RNA. Wastewater samples have been utilized to identify several pathogenic human viruses, and consequently, it has gained attention for assessing population-level trends of SC-2 infections.

Detection of SC-2 in wastewater was reported in numerous studies, including North America [8,9,10,11], Europe [12,13,14,15,16], Asia [17,18], and Oceania [19], with feasibility addressed in the review by Mousazadeh et al. [5]. These studies describe a range of sample concentration and viral RNA recovery approaches, followed by RT-qPCR to determine the viral load. These proof-of-concept studies indicate that wastewater monitoring can be used as an early detection tool for health authorities [20,21].

The emergence of SC-2 variants that have the potential for increased transmissibility and/or immune response evasion raised an urgent need for targeted surveillance of circulating lineages. SC-2 variant B.1.1.7 (https://nextstrain.org/sars-cov-2/ (accessed on 1 May 2022), Alpha variant) was first identified in the UK during December 2020, where it became the dominant SC-2 variant within four weeks [6]. The genome of variant B.1.1.7 contains 23 mutations, some of which are associated with increased transmissibility, which can lead to higher viral loads in infected patients, and subsequent increased mortality [22]. The dynamic shifts in SC-2 variant dominance require enhanced surveillance of emerging lineages, such as B.1.1.7, to facilitate appropriate response by public health authorities. Whole genome sequencing (WGS) is considered the most comprehensive method for identification of emerging variants [23,24]. However, its use is time-consuming, expensive, requires highly skilled personnel, and cannot be readily scaled up to accommodate a large number of samples. Methods based on RT-qPCR, which include a “drop-out” signal and are available in commercial kits (such as the Thermo Scientific TaqPath COVID-19 diagnostic kit) or the method published in a recent study [25], are cost-effective, rapid, and can be readily scaled up in any molecular biology laboratory. In this test, the A19/A20 (“WT”) variant gives a positive signal in three parallel reactions, each targeting a different region within the viral genome. When an Alpha variant sample is tested, one reaction is negative, and the overall test result is therefore indicative of the presence of this variant in the sample. This approach has a major limitation, which stems from the issues associated with absence of signal. Such a “drop out” can result from low sensitivity or inhibition and may not necessarily reflect the presence of the target mutation. Furthermore, in the case of the Thermo Scientific kit, the 69-70 mutation, which is the cause of the “drop out” effect, is not unique to variant B.1.1.7. and cannot be considered as a reliable indication of this variant. Other commercial kits that target specific SC-2 mutations are more specific but require complicated interpretation and, in some cases, multiple tests per sample to determine the sample identity (https://www.kogene.co.kr/eng/ (accessed on 1 May 2022), https://www.seegene.com/ (accessed on 1 May 2022), en.vircell.com (accessed on 1 May 2022)).

The third morbidity wave of COVID19 in Israel started in mid-November 2020. In mid-December 2020, the first cases of SC-2 Alpha variant were detected in Israel [26]. As a result, there was a shift in the SC-2 variant dominance from A19/A20 (“WT” Wild-type, Wuhan strain) to B.1.1.7 (Alpha) variant in Israel. The national vaccination campaign (using the BNT162b2 Pfizer vaccine) started at the end of December 2020, and by the end of March 2021, 56% and 51% of the total population were vaccinated with the first and the second doses, respectively (https://datadashboard.health.gov.il/COVID-19/general (Hebrew, accessed on 1 May 2022)).

In a previous report, we described the development of a PCR assay that identifies a mutation unique to variant B.1.1.7. and showed that it can be used as a reliable marker for its presence of the unknown samples [27]. Here, we combined this assay with a complementing test so that the relative load of either B.1.1.7. or non-B.1.1.7. RNA in an examined sample can be evaluated. This study demonstrates the use of a rapid test to monitor the SC-2 variant dynamics on a national scale in real time by continuous sampling of wastewater.

## 2. Materials and Methods

### 2.1. Wastewater Sampling and Processing

The wastewater sampling sites (*n* = 13) selected for this study were collected from various regions of Israel covering more than 55% of Israel’s population. These samples were collected from wastewater treatment plants (WWTP) between December 2020 and March 2021 (*n* = 117 samples). In each sampling cycle, 250 mL of untreated wastewater were collected every 30 min for 24 h by external automatic composite samplers. Samples were then transported to the laboratory under cooled conditions (4 °C). The sample material was concentrated as previously described [19], with minor changes. A duplicate homogeneous fraction of the wastewater sample (25 mL) was centrifuged (4696 *g*) for 5 min at 4 °C, and 20 mL of the supernatant was collected into 50 mL tube containing 0.26 M of MgCl_2_. The tubes were gently stirred for 5 min. Each sample duplicate was then passed through 0.45 μm pore-size, 47 mm diameter electronegative MCE membranes (Merck Millipore Ltd. Darmstadt, Germany) by peristaltic pumping. The membrane was transferred into a 50 mL tube containing 3 mL external lysis buffer (NucliSENS easyMAG, Marcy-l’Étoile, France). The tube with the membrane was gently stirred with the membrane inside for 30 min for virus inactivation and removal. The membrane was removed, and the lysis buffer containing the viral RNA was taken for RNA extraction.

### 2.2. Viral RNA Extraction

Total nucleic acid (NA) were extracted using the NucliSENS easyMAG system (Biomerieux, Marcy-l’Etoile, France) following the manufacturer’s instructions. Briefly, 3 mL of lysis buffer containing the inactivated virus concentrated sewage were extracted using the easyMAG machine. Extracted NA were eluted using 55 μL elution buffer and stored at −70 °C.

### 2.3. Primers and Probes

Primers and probe for the OC43 spike control reaction were from Dare et al. [28]. Primers and probe for the SC-2 Envelope (E) gene reaction were based on the sequences published by Corman et al. [29], with the modifications detailed in Table 1. The primers for the SC-2 nucleocapsid (N) gene reaction were based on the CDC nCov-N1 reaction (https://www.cdc.gov/coronavirus/2019-ncov/lab/rt-pcr-panel-primer-probes.html, (accessed 1 May 2022)), with the modification detailed in Table 1. The B.1.1.7 specific reaction was based on the CDC nCoV-N1, with modifications described previously [27]. The WT-specific N1 reaction was termed N_WT_, and the Alpha-specific reaction was termed N_B.1.1.7_ hereafter.

### 2.4. RT-qPCR

RT-qPCR mix was prepared using the Meridian (formerly Bioline) SensiFast one-step mix (https://www.bioline.com/sensifast-probe-no-rox-one-step-kit.html, accessed on 1 May 2022, Waltham, MA, USA), with the addition of PCR-grade Bovine Serum Albumin (BSA) at a final concentration of 300 nM. The reaction was run using Bio-Rad CFX96 instrument (https://www.bio-rad.com/en-us/product/cfx96-touch-real-time-pcr-detection-system?ID=LJB1YU15, accessed on 1 May 2022, Hercules, CA, USA). The cycling conditions were as follows: 45.0 °C for 15 min, 95.0 °C for 2:20 min, 45X (95.0 °C for 5 s, 60.0 °C for 42 s). Fluorescence was read at 60 °C on each cycle. Analysis of the results were performed using the Bio-Rad CFX Maestro software (https://www.bio-rad.com/en-us/product/cfx96-touch-deep-well-real-time-pcr-detection-system?ID=LZJTUJ15, accessed on 1 May 2022, Hercules, CA, USA).

### 2.5. Design and Generation of In Vitro Transcribed Standard RNA Templates

In-vitro transcribed RNA molecules were used as control templates to maintain uniformity and to establish the reaction analytical limit of detection (LOD). Generation of RNA templates was performed using the Megascript kit (Thermo Fisher, https://www.thermofisher.com/order/catalog/product/AMB13345#/AMB13345, accessed on 1 May 2022, Waltham, MA, USA), which allows in-vitro synthesis of RNA from DNA template using the T7 phage DNA-dependent RNA polymerase. Regions flanking the target sequences of each reaction were amplified using specific primers fused to the T7 phage minimal promoter sequence and were then used as template for the RNA synthesis. The resulting RNA templates were purified using the PSS magLEAD instrument (http://www.pss.co.jp/, accessed on 1 May 2022, Chiba, Japan). The RNA yield was determined using the NanoDrop spectrophotometer (https://www.thermofisher.com/i, accessed on 1 May 2022, Waltham, MA, USA) and used to generate standard curves for each reaction. The analytical limit of detection (LOD) was determined using serial dilutions of the RNA targets, with the multiplex mix. This calibration was then used to calculate the number of copies detected in wastewater samples.

### 2.6. Calculation of the Normalized Viral Load Value in Wastewater Samples

In order to compare the viral load as measured by qPCR, in different catchment points that have different flow rates and are draining regions with varying population size, we used a normalized viral load (NVL) conversion that takes into account these variables [30]. Using standard curves generated with the in vitro transcribed standard RNA templates, qPCR Cq values of the examined samples were converted into viral RNA copies. These values were then used for the NVL calculation according to the following equation:

$$1\mathrm{NVL}\;\left(\mathrm{RNA}\frac{\mathrm{copies}}{\mathrm{population}\times \mathrm{day}}\right)=\frac{\mathrm{RNA}\;\mathrm{copies}\;\times \;\mathrm{Flow}\;\mathrm{rate}}{\mathrm{Population}\;\mathrm{per}\;1000}$$

where RNA copies per liter of wastewater is the converted from Cq value, flow rate is the measured flow rate (liter per day) as measured in the specific catchment point, and population per 1000 is the size of the population in thousands, which reside in the region that the specific catchment point is collecting.

Calculation of the average NVL for each region was performed by determining the average NVL from each month (December, January, February, and March) for each WWTP. Then, the average NVL values from all the points of each region were summarized for each month. NVL values based the copy number derived from the E gene reaction were calculated to determine the total NVL of SC-2 in the sample pool. The percentage of the A19/B19 RNA and the B.1.1.7 RNA were determined by the NVL values of N_WT_ reaction and N_B.1.1.7_, respectively.

### 2.7. Whole Genome Sequencing and Bioinformatic Analysis

The procedure of library construction and WGS analysis was described in detail previously [27]. Briefly, the SC-2 genome was amplified using the Illumina COVID-seq kit, and libraries were prepared using the NexteraXT kit (https://emea.illumina.com/, accessed on 1 May 2022). Resulting Fastq sequence files that passed QC were compared to SC-2 reference genome (NC_045512.2) and analyzed for the presence and frequency of mutations that are associated with the B.1.1.7 variant. All genomic sequences used in this study were deposited in the GISAID database (https://www.gisaid.org/, accessed on 1 May 2022).

### 2.8. Ethical Statement

The sequencing of clinical samples was conducted according to the guidelines of the Declaration of Helsinki and was approved by the Institutional Review Board of the Sheba Medical Center (7045-20-SMC). Patient consent was waived as remains of clinical samples were used, and the data was anonymized during the analysis.

## 3. Results

### 3.1. Development of the Positive/Negative B.1.1.7 Variant Reaction

The reaction that specifically detects the B.1.1.7 variant was described previously [27]. Briefly, a primer that binds specifically to the D3L substitution was designed and used, designated as 28257 N D3L VOC (highlighted in Table 1). This mutation is almost exclusive to variant B.1.1.7 and is not currently associated with any other major SC-2 variant, as evident from the NextStrain global mutation analysis (Figure S1A). In order to perform a reciprocal reaction that detects only the WT lineage, a reciprocal primer that binds exclusively to the WT sequence was designed. The sequences and primer binding sites are illustrated in Figure S1B. In order to monitor the on-going spreading of variant B.1.1.7 in Israel, each sample was tested at least twice: once with the N_B.1.1.7_ assay and once with its reciprocal N_WT_ reaction. For continuous monitoring of the variant circulation in wastewater, each sample was tested with a multiplex containing the E, N_WT_, and OC43 reactions and again with the E and N_B.1.1.7_ reactions. This enabled us to evaluate the overall SC-2 circulation in the examined wastewater treatment plants (WWTP) and, at the same time, determine the percentage of WT versus B.1.1.7 variants in that WWTP.

### 3.2. Development of RT-qPCR Assay for Detection of SC-2 in Environmental Samples

The designed primers were required to detect the presence of SC-2, to distinguish between the WT and variant B.1.1.7 strains, and to confirm the integrity of the sample preparation. In order to meet these requirements, the following triplex qPCR assay was developed. An inclusive SC-2 reaction targeting a conserved sequence within the SC-2 envelope (E) gene was used as a control for the presence or absence of SC-2 RNA in the sample. A second reaction targeting the N gene B.1.1.7 variant-associated mutation D3L was used as the differential reaction where the variant-specific primer was altered depending on the reaction specificity. A third reaction targeting the hCoV OC43 was used as an internal spiked control to evaluate the pre-extraction procedure integrity.

The specificity of the Alpha-specific reaction was already established previously [27]. In order to evaluate its selectivity with WW samples, 19A/19B and B.1.1.7 samples were examined each using either the N_WT_ reaction or the N_B.1.1.7_ reaction. Figure S2 shows representative reactions in which the 19A/19B sample was positive for both the E and N targets when using the N_WT_ reaction but only for the E target when using the N_B.1.1.7_ reaction. The B.1.1.7 sample gave the reciprocal result, i.e., positive for the E target only with the N_WT_ reaction and positive for both targets with the N_B.1.1.7_ reaction (Figure S2). The components of the three reactions were then combined together, and the sensitivity of each duplex was tested using in vitro synthesized RNA targets. Serial dilutions of the RNA targets were spiked into wastewater extraction to simulate an authentic sample background medium. The analytical limit of detection (LOD) derived from the dilutions testing was determined as follows: 35 copies per reaction for the E reaction, 12.9 copies for the N_B.1.1.7_ reaction, 13.5 copies for the N_WT_ reaction, and 28 copies for the OC43 reaction (Figure S3). For each test, the following positive controls were used: WT SC-2 RNA, B.1.1.7 RNA, and CoV OC43 RNA. SC-2-negative WW extraction was used as negative control. For quantitative analysis, serial dilutions of in vitro synthesized RNA targets were used.

### 3.3. Analysis of SC-2 Variants Dynamics Using the Differential Assay

**Figure 1:**
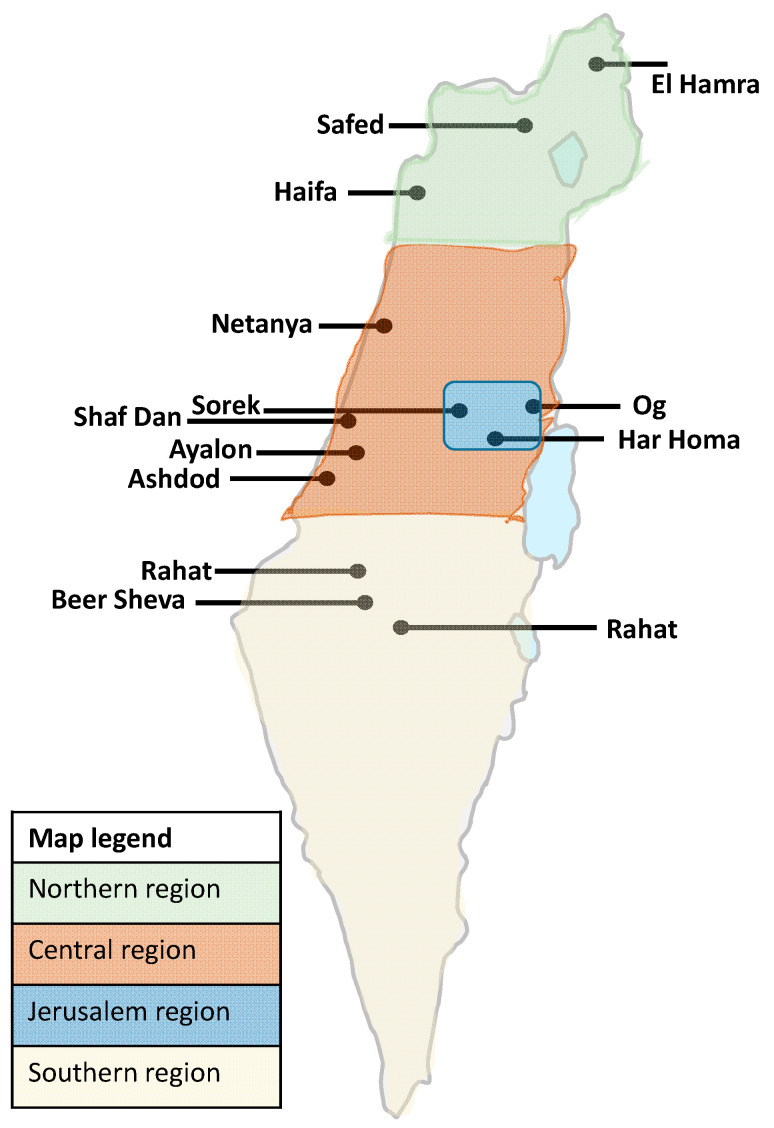
Location of wastewater treatment plants (WWTPs) included in this study. The WWTPs were clustered into four regions, each labelled in a different color, as detailed in the map legend. The calculated viral load and relative variant prevalence for each region was based on the average values of the WWTPs in each region.

Wastewater monitoring of SC-2 was performed from December 2020 to March 2021 in 13 WWTPs across Israel (Figure 1). WWTPs population catchment area is estimated at about 5.2 million people, which is over 55% of Israel’s population (Table 2). The WWTPs were divided into four groups: north, central, Jerusalem, and south. In each regional group, 3–4 WWTPs were sampled periodically at least once a month. Each WWTP was sampled between 6 and 22 times depending on the sampling resources availability and the site accessibility. Each RNA extraction from each time point was tested at least twice. For each WWTP, the average normalized viral load (NVL) value was calculated from the repeats of the SC-2 E reaction. When applicable, the standard deviation was calculated and plotted on the representing graph. Analysis of the SC-2 dynamics in the northern region showed a mixed trend, where a slight decrease of the total normalized viral load (NVL) was observed in the El Hamra and Safed WWTPs, and a sharp decrease was evident in the Haifa WWTP. Turnover of the variant dominance from WT to B.1.1.7 was completed by the end of February 2021 (Figure S4A). Monitoring of the four WWTPs of the central region showed a similar dynamic trend, with total NVL peaking in 21 January and slightly decreasing by March. As was in the northern region, by the beginning of March, nearly all samples contained only the variant B.1.1.7 (Figure S4B). The total NVL values in the Jerusalem region WWTPs showed a similar course, in which the total NVL increased until January and then decreased by approximately 90% by March. The variant prevalence switched from 100% WT in December 2020 to 100% B.1.1.7 by the beginning of March 2021 (Figure S5A). Interestingly, while the variant dynamics in the southern region were similar to those in the other three regions, the overall NVL showed a slight increase from December 2020 until the end of March 2021 (Figure S5B). Comparison of the variant prevalence in February 2021 showed that the only WWTP in which the WT prevalence was above 46% was El Hamra (99%). In Safed, Ayalon, and Rahat, the B.1.1.7 variant consisted between 92% and 99% (Figure S6).

**Figure 2:**
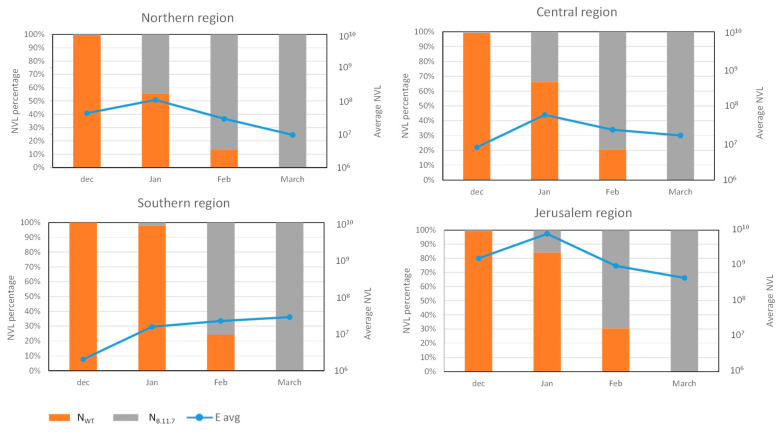
Wastewater SC-2 averaged normalized viral load in different regions of Israel. The blue line represents the averaged amount of E gene NVL in each month in the different regions. The orange and gray bars show the average percentage of N_wt_ gene and N_B.1.1.7_ gene in every month in different regions of Israel.

In order to identify general trends in the variant dynamics, we clustered the results from the WWTPs of each region and examined the dynamics by region, as shown in Figure 2 and detailed in Table 2.

For each regional group, the NVL values from all WWTP sites within that group were used to calculate the regional average value. The average values obtained for each point were summarized for each region, and the percentage of the SC-2 variant was determined (Figure 2). The variant turnover was similar in all four regions, switching from 100% WT in December 2020 to 100% variant B.1.1.7 by March 2021. The shift was somewhat delayed in the southern region, where in January, the WT prevalence was almost 100% compared to 15–45% in the other regions (Figure 2). However, the shift in the southern region was faster compared to the Jerusalem region at 75% and 70% variant B.1.1.7 prevalence, respectively (Figure 2). Although the switch from WT to B.1.1.7 was somewhat different in each region, all of them were 100% B.1.1.7 by mid-March (Figure 2, Figures S4 and S5). Comparison of The total NVL showed a moderate but clear decrease in the northern, central, and Jerusalem regions from January to March, while in the southern region, the NVL increased and remained constant during that period (Figure 2). Detailed data for each WWTP site, including the calculated NVL values, are presented in Figures S4 and S5.

### 3.4. Variant Dynamics with Respect to Clinical Samples Sequencing and Vaccination Status

**Figure 3:**
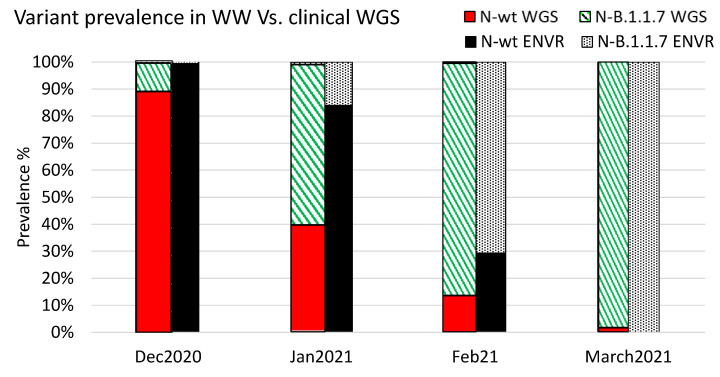
Relative variant prevalence in WW samples and sequenced clinical samples. The relative variant prevalence is shown between December 2020 and March 2021 for clinical samples and WW samples. Clinical samples were sequenced and classified using the Illumina COVID seq platform as described previously [27]. The WW values were calculated based on the average values obtained from all catchment sites in each month.

In order to assess the correlation between WW-based variant dynamics and sequenced clinical samples, the percentage of B.1.1.7-positive samples was calculated from the total number of samples that were sequenced and analyzed in the Israel Central Virology Laboratory (CVL) between December 2020 and March 2021 [27]. The results were then compared to the average percentages of B.1.1.7 and WT from all WWTP tested in the study. The average percent of either WT or B.1.1.7 was then plotted against the percentage of B.1.1.7-negative or B.1.1.7-positive clinical samples, respectively. As shown in Figure 3, on December 2020, 11% of the sequenced samples were identified as B.1.1.7, while only 1% of the total viral load calculated for WW samples was identified as such.

During January, 60% of the sequenced samples were classified as B.1.1.7, and 16% of the WW samples were classified as such by B.1.1.7-speficic qPCR. In February 2021, 70.7% of the WW samples were identified as containing the B.1.1.7 variant, and 87% of the sequenced samples were classified as B.1.1.7. By the end of March 2021, all WW samples were only positive for B.1.1.7 (no WT SC-2 was detected), and 98% of the sequenced clinical samples were identified as B.1.1.7 (Figure 3). Notably, many of the clinical samples were not selected randomly for sequencing and may therefore somewhat skew the overall results. Nevertheless, these data indicated a clear correlation between the sequencing results and the dynamics inferred from WW examination.

During December 2020, when the Alpha variant was introduced into Israel, a national vaccination campaign was rolled out, using the two-dose BNT162b2 administration (Pfizer). By comparing the wastewater normalized viral load (WW NVL) of each region, we evaluated the possible effect of vaccination on the circulation of SC-2 in wastewater. 

**Figure 4:**
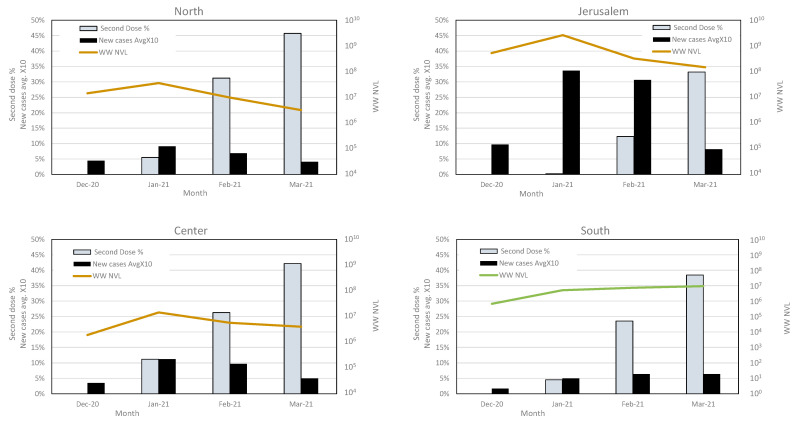
Average of second dose vaccination and new cases rate, compared with wastewater viral load in each region. The second-dose vaccination rates, new SC-2 infection cases and total wastewater normalized viral load (WW NVL) were compared between December 2020 and March 2021 for each region. For clarity of presentation, the percentage of average new cases was multiplied by 10. The WW NVL was calculated as detailed in the Methods section. The vaccination and clinical cases data are from the Israel Ministry of Health website (https://datadashboard.health.gov.il/COVID-19/general, accessed on 1 May 2022).

The total WW NVL were similar in the northern, central, and Jerusalem regions, with a NVL peak on January and a gradual decrease towards March, concurrent with the increase in second-dose vaccination rates. This trend was accompanied by a decrease in the new cases reported between January and March (Figure 4 and Table 3). The average number of new cases in the northern, central, and southern regions was comparable, reaching approximately 1% in January 2021 and decreasing to 0.4–0.6% by March. The average number of new cases in the Jerusalem region was significantly higher, ranging from 3.4% in January to 0.8% in March (Table 3). Although the vaccination rates in the southern region were similar to the other regions, the number of new cases in that region increased, and the total WW NVL elevated accordingly (Figure 4 and Table 3). The decrease in total WW NVL and reported new cases in all other regions was parallel to the variant turnover, from WT to B.1.1.7 (Figure 2 and Figure 4).

## 4. Discussion

From the incursion of the SC-2 into Israel, until February 2021, the dominant SC-2 clade in Israel was the Wuhan 19B/20B. The Alpha variant was first identified in Israel at the end of December 2020 [27], and by March 2021, it consisted of more than 90% of randomly sequenced clinical samples (Israel Ministry of Health, data not published). Environmental surveillance proved as an important tool in monitoring the COVID-19 pandemic in populations and entire large geographic regions [21,31,32]. However, information on real-time dynamics of SC-2 variants on a national scale is currently insufficient. In this report, we describe the real-time prevalence of circulating SC-2 A19/B19 (Wuhan strain) and B1.1.7 variant, during a period of 4 months, across Israel. Some recent studies of SC-2 variants surveillance relied on the use of genomic sequencing or PCR detection of mutations that are not specific to a particular variant [33,34]. Other studies describe environmental surveillance using a combination of variant-specific and variant-nonspecific reactions [35,36]. Peterson et al. [36] describe the use of six reactions to indicate the presence of Alpha, Beta (B.1.351) or Gamma (P1) variant but not distinguish between them. The authors examined WW from five major cities as well as rural communities, determining the prevalence of the three mutations in each site. While the Alpha variant was verified by the specific N D3L-directed reaction, as we performed in our study, the other two reactions detected the spike 69–70 and N501Y mutations. These are characteristic but are not unique to the alpha variant [37]. Although the authors suggest that identifying these mutations may indicate the presence of Beta or Gamma variants, there was no definite evidence in the data presented that this was indeed the case. Furthermore, the need to run a separate assay for each mutation and the absence of an inclusive control (the “WT” reaction was run for each sample separately, in parallel with the “mutation” reaction) render this approach cumbersome. In our study, we combined an inclusive reaction (E-sarbeco) with the Alpha-specific reaction (D3L) and a control reaction (MS-2 phage spiking) to enable rapid and specific screening of a large number of samples.

The comparative analysis shows that the Alpha variant emerged in parallel on different sites in an apparently independent pattern. The first sites in which alpha variant was dominant by mid-February were remote from each other (Safed (Safed, Israel), Ayalon (Tel-Aviv, Israel, and Rahat (Rahat, Israel), Figure S4). This pattern further supports the assumption of independent Alpha variant spread. The different variant dynamics during January–February may reflect different infection events at the community level or simply result from sampling bias, as not all WWTPs were equally accessible for sampling. The complete turnover of all regions in March is consistent with the global variant dynamics, where the Alpha variant overtook the WT original SC-2 strain [37]. Comparison of the WW samples analysis to sequenced clinical samples showed an apparent preceding of the clinical samples turnover (Figure 3). This was likely due to the sequencing prioritization in December 2020 and January 2021, which included severe patients, incoming international travelers, samples with unusual PCR results, and other unique cases, all of which did not represent a random selection. Nevertheless, the overall variant prevalence, as reflected in clinical samples sequencing (WGS), was in agreement with the WW sampling results. In this respect, WW sampling may be more representative than individual sequencing since it is less prone to sampling bias, providing a pooled, heterogeneous sample material. These results indicated that WW monitoring could indeed be used as a good surveillance approach for identifying circulating viral variants. 

Analysis of the variant dynamics with respect to population vaccination status showed a correlation between the increase in second-dose vaccination rates and the decline in total WW, NVL, and reported new clinical cases. This occurred regardless of the dominant variant. It is important to note that although the NVL dynamics in the southern region indicated an increase rather than a decrease, the calculated viral load was comparable to that of the northern and central regions and less than the Jerusalem region. The viral dynamics described in our study are consistent with the recent findings described by Yaniv et al. [35], which showed the turnover of the SC-2 variant during the beginning of 2021 in the city of Be’er Sheva. In a proceeding publication, the authors demonstrated that increased vaccination rates correlate with WW NVL decrease [37]. The authors conducted a thorough monitoring of the variant dynamics in Be’er Sheva and showed that the NVL increased from November 2020 to March 2021 and then started to decrease, following the increase in the second-dose vaccination rate [38]. We expanded this examination to 13 different sites across Israel and found a similar overall change with local differences between different sites, with evident correlation between the new cases and the NVL values.

In this study, which focused on the SC-2 WT-Alpha prevalence, we showed how a real-time and retrospective monitoring of WW could be used to establish the variant dynamics and reflect the population-level response to a national scale vaccination campaign over a period of 4 months. 

Our data show a clear shift in the viral RNA circulation following the vaccination campaign, thereby demonstrating the importance of wastewater surveillance as an epidemiological tool. Previous reports were limited in the number of sites examined and mostly used sequencing to identify the circulation of variants of interest [39,40]. While sequencing provides complete information on the examined sample, it is expensive, not readily amendable for high throughput, and it is limited by the sample quality. We recently demonstrated that variant-specific PCR can determine SC-2 variant identity in cases of poor sample quality where sequencing fails [41]. Here, we show that variant-specific PCR can be a valuable tool to rapidly identify variant dynamics in WW in a cost-effective manner, which in turn enables a large-scale screening of WW samples to obtain important epidemiological information. Such an approach may be useful in current and future events of variant switch, as is happening now in many countries, with the BA.1 and BA.2 variants spreading (Bar-Or and Erster, manuscript in preparation). In addition to serve as an early warning system for the spreading of new variants, it allows a retrospective analysis of variant dynamics and vaccination response, thereby contributing to our understanding of the COVID19 progression and evolution.

## Figures and Tables

**Table 1:** Primers and probes used in this study.

Primer Name	Sequence (5′->3′) and Modifications
Corona-OC-43-F	CGATGAGGCTATTCCGACTAGGT
Corona-OC-43-R	CCTTCCTGAGCCTTCAATATAGTAACC
Corona-OC-43-P	TEXASRed-TCCGCCTGGCACGGTACTCCCT-BHQ2
E-Sarbeco F1b	GTTAATAGCGTACTTCTTTTTCTTGC
E_Sarbeco_R2	ATATTGCAGCAGTACGCACACA
E_Sarbeco_P1	FAM-6-ACACTAGCCATCCTTACTGCGCTTCG-BHQ1
28258D3L WT Fwd	AAACGAACAAACTAAAATGTCTGAT
28257 N D3L VOC Fwd	TAAACGAACAAACTAAATGTCTCTA
2019-nCoV_N1-R	TCTGGTTACTGCCAGTTGAATCTG
2019-nCoV_N1-P	Hex-ACCCCGCATTACGTTTGGTGGACC-BHQ1
OC43 pT7 29363F	**TAATACGACTCACTATAGGG** GGTACTGGTACAGACACAACAGAC ^1^
OC43 29960 Rev	CCACCAAAATTCTGATTAGGGCCTC
T7 nCoV 26067 Fwd	**TAATACGACTCACTATAGGG** GTACAGACACTGGTGTTGAACATG ^1^
nCoV 26441 Rev	CTCTAGAAGAATTCAGATTTTTAACACG
pT7 nCoV 28225 F	**TAATACGACTCACTATAGGG** GAAGACTTTTTAGAGTATCATGAC ^1^
COV19 29577 Rev	CCATCTGCCTTGTGTGGTCTGCATG

^1^ The T7 minimal promoter fused to the 5′p of the primer is in bold letters.

**Table 2:** Details of the wastewater treatment plants (WWTPs) sampled from December 2020 until March 2021.

WWTP Name	Population	Flux (Cubic Meters Per Day)	Region
El Hamra	21,504	2240	North
Haifa	583,147	100,000	
Safed	36,933	4400	
Netanya	291,981	39,000	Center
Shafdan	2,291,901	390,000	
Ayalon	375,649	54,000	
Ashdod	225,939	31,100	
Sorek	873,267	108,167	Jerusalem district
Og	180,000	26,586	
Har Homa	31,250	5000	
Be’er Sheva	272,448	39,500	South
Arara	19,328	1700	
Rahat	77,335	4500	
Total	5,280,682	806,193	Israel (9,291,000 population)

**Table 3:** Anti-SARS-CoV-2 vaccination rates in the different regions of Israel during December 2020–March 2021.

Month	First Dose Percent			
	North	Central	Jerusalem	South
December 2020	2.30%	6.50%	1.30%	0.00%
January 2021	27.00%	28.70%	13.50%	19.00%
February 2021	46.10%	41.40%	21.20%	35.30%
March 2021	58.30%	53.50%	41.90%	48.50%
	Second Dose Percent			
December 2020	0.00%	0.00%	0.00%	0.00%
January 2021	5.50%	11.20%	0.20%	4.50%
February 2021	31.30%	26.30%	12.30%	23.50%
March 2021	45.70%	42.20%	33.20%	38.50%
	AVG 14 Days of New Cases			
December 2020	0.40%	0.30%	1.00%	0.20%
January 2021	0.90%	1.10%	3.40%	0.50%
February 2021	0.70%	1.00%	3.10%	0.60%
March 2021	0.40%	0.50%	0.80%	0.60%

## Data Availability

Data regarding population vaccination and new positive SC-2 cases was obtained from the publicly available Israel Ministry of Health website at: https://datadashboard.health.gov.il/COVID-19/general (accessed on 1 May 2022). The genomic sequencing data was deposited in the GISAID database (https://www.gisaid.org/, accessed on 1 May 2022).

## Supplementary Materials

The following supporting information can be downloaded at: https://www.mdpi.com/article/10.3390/v14061229/s1, Figure S1: Design of a differential WT/alpha reaction; Figure S2: Representative amplification curves of the duplex SC-2 reactions; Figure S3: Standard curves of the SC-2 assay reactions; Figure S4: SC-2 total load and variant dynamics in the WWTPs of the northern (A) and central (B) regions; Figure S5: SC-2 total load and variant dynamics in the WWTPs of the Jerusalem district (A) and Southern (B) regions; Figure S6: Average variant prevalence in each WWTP during February 2021.
