# Supplementary material for: National Scale Real-Time Surveillance of SARS-CoV-2 Variants Dynamics by Wastewater Monitoring in Israel
Source: Viruses. 2022 Jun 6;14(6):1229. doi: 10.3390/v14061229 (PMC9227326; doi:10.3390/v14061229)
Supplement: Supplementary file 1 [file viruses-14-01229-s001.zip › viruses-1731717-supplementary.pdf]

SUPPLEMENTARY MATERIAL

National Scale Real-Time Surveillance of SARS-CoV-2 Variants Dynamics by Wastewater Monitoring in Israel

Itay Bar-Or [1,†], Victoria Indenbaum [1,†], Merav Weil [1], Michal Elul [1], Nofar Levi [1], Irina Aguvaev [1], Zvi Cohen [1], Virginia Levy [1], Roberto Azar [1], Batya Mannasse [1], Rachel Shirazi [1], Efrat Bucris [1], Orna Mor [1,2], Alin Sela Brown [1], Danit Sofer [1], Neta S. Zuckerman [1], Ella Mendelson [1,2], and Oran Erster [1,*]

[1] Central Virology Laboratory, Israel Ministry of Health, Chaim Sheba Medical Center, Ramat Gan 5262000, Israel; itay.baror@sheba.health.gov.il (I.B.-O.); viki.indenbaum@sheba.health.gov.il (V.I.); merav.weil@sheba.health.gov.il (M.W.); michal.elul@sheba.health.gov.il (M.E.); nofar.levi@sheba.health.gov.il (N.L.); irina.aguvaev@sheba.health.gov.il (I.A.); zvi.cohen2@sheba.health.gov.il (Z.C.); virgini.levy@sheba.health.gov.il (V.L.); roberto.azar@sheba.health.gov.il (R.A.); batya.mannasse@sheba.health.gov.il (B.M.); rachel.shirazi@sheba.health.gov.il (R.S.); efrat.bucris@sheba.health.gov.il (E.B.); orna.mor@sheba.health.gov.il (O.M.); alinselabrown@gmail.com (A.S.B.); danit.sofer@sheba.health.gov.il (D.S.); neta.zuckerman@sheba.health.gov.il (N.S.Z.); ella.mendelson@sheba.health.gov.il (E.M.)

[2] Sackler Faculty of Medicine, School of Public Health, Tel-Aviv University, Tel-Aviv 69978, Israel

* Correspondence: oran.erster@sheba.health.gov.il

† These authors contributed equally to this work.

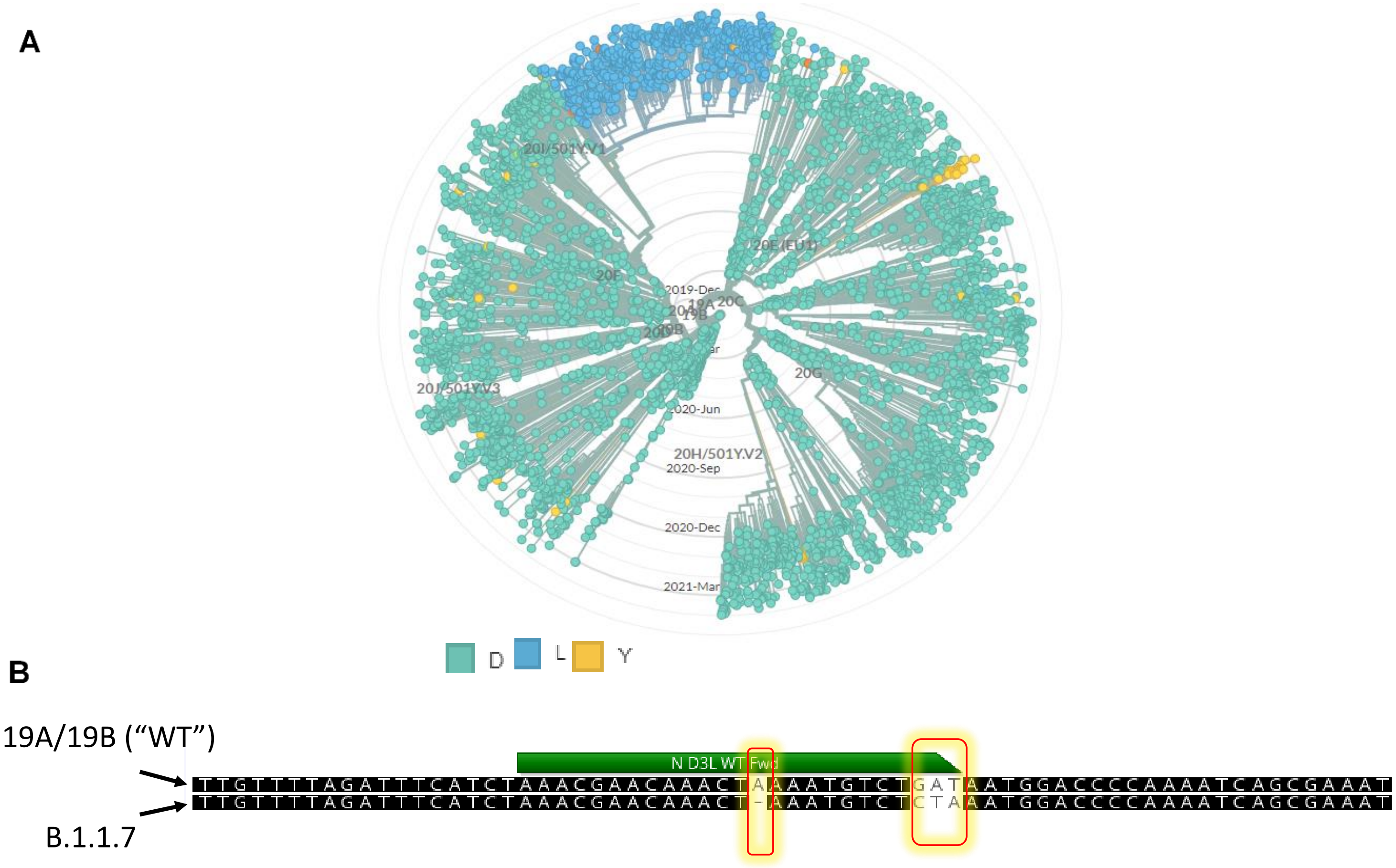

Figure S1. **Design of a differential WT/alpha reaction.** (A) NextStrain global Analysis of the Nucleocapsid (N gene) sequence showing the uniqueness of the D3L mutation to alpha variant. The dendrogram shows the clades that contains the substitution from D (grey) to L (blue) or Y (orange) in positon 3 of the N protein sequence. (B) Alignment of the specific primer-binding region. The mutations are highlighted.

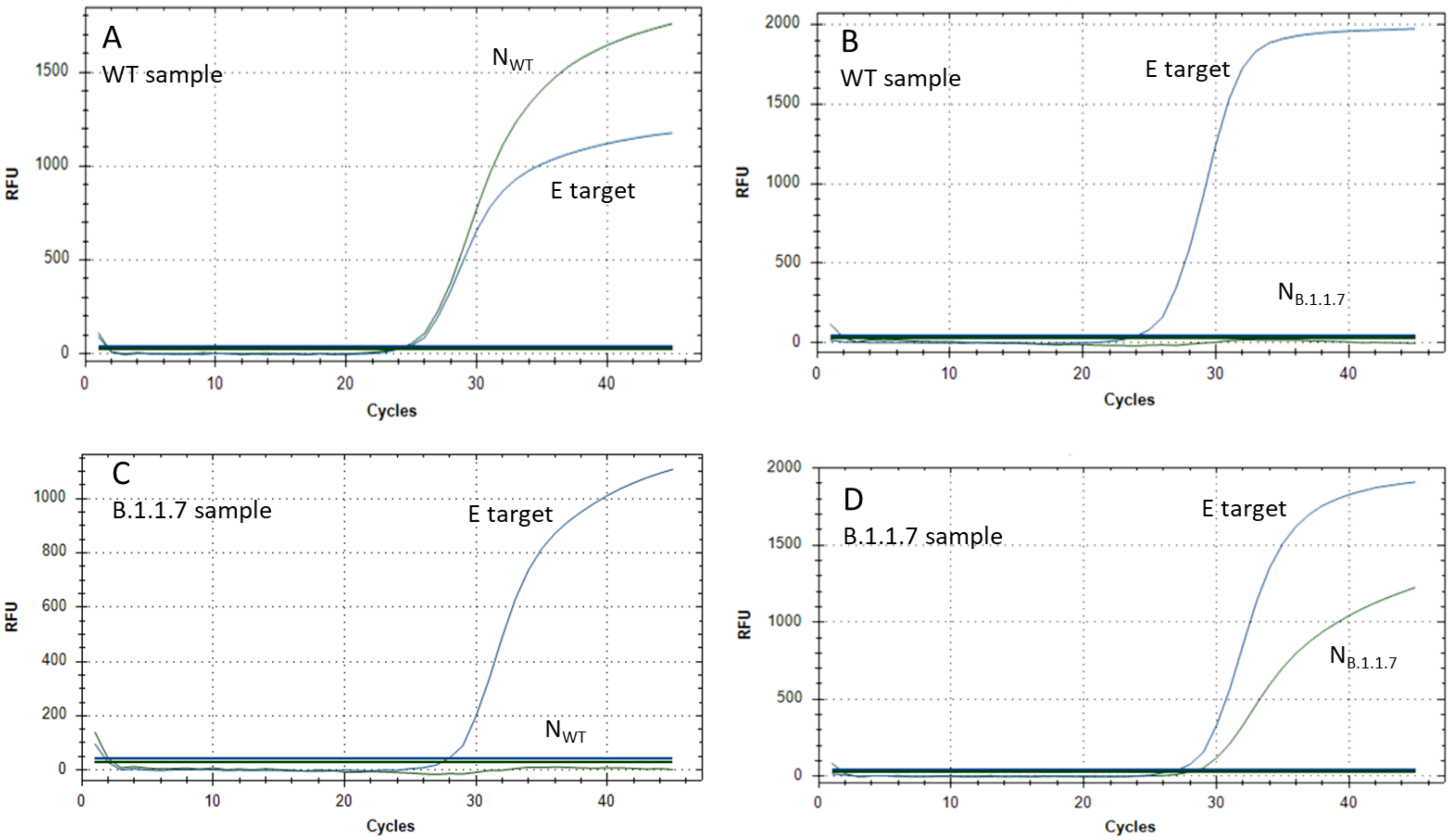

Figure S2. **Representative amplification curves of the duplex SC-2 reactions**. Each of the multiplex reactions was used with either A19/B19 ("WT") sample or B.1.1.7 (alpha) sample. the tested samples were previously sequenced and their classification was established. (A) WT reaction with WT sample. (B) Alpha reaction with WT sample. (C) WT reaction with alpha sample. (D) Alpha reaction with alpha sample. WT reaction: Multiplex detecting the E and N_{WT} targets. Alpha reaction: Multiplex detecting the E and $N_{B.1.1.7}$ targets.

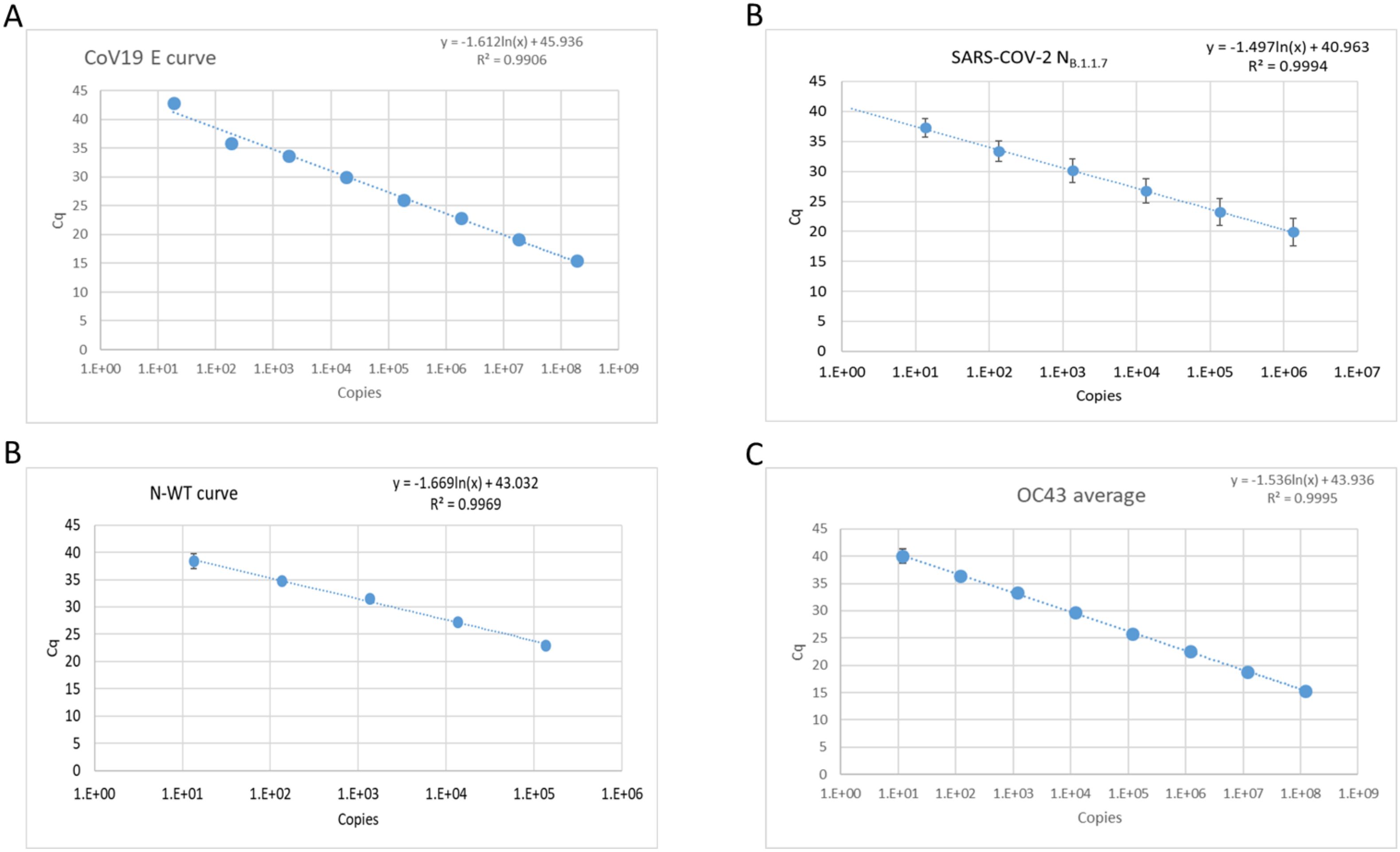

Figure S3. **Standard curves of the SC-2 assay reactions**. The reaction mix was tested with *In vitro* transcribed RNA molecules containing the assay target sequences. Serial dilutions of each target were tested in triplicates and the resulting regression formula was calculated. (A) E-sarbeco reaction curve. (B) $N_{B.1.1.7}$ reaction curve. (C) N_{WT} reaction curve. (D) CoV OC43 reaction curve. The R^2 value and the regression formula are shown for each curve.

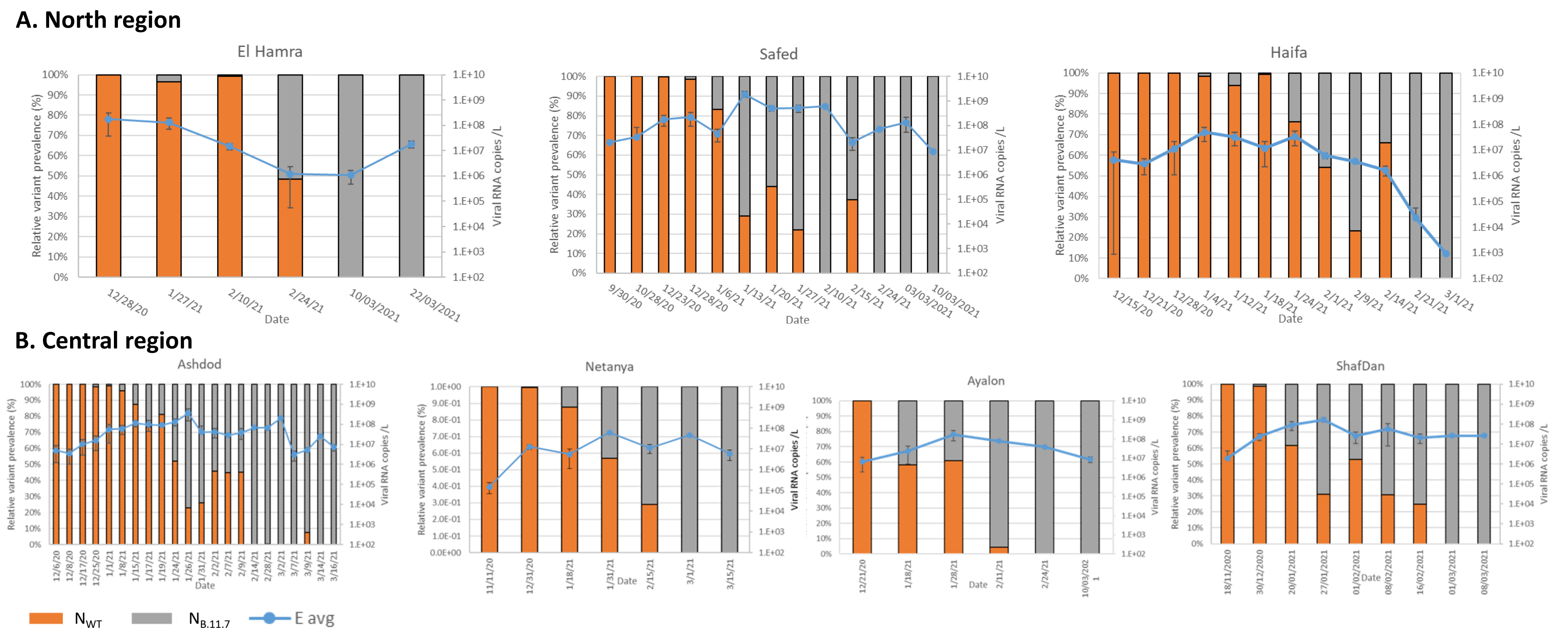

Figure S4. SC-2 total load and variant dynamics in the WWTPs of the northern (A) and central (B) regions. The E gene reaction (line with markers) represent the total SC-2 viral RNA copies (left Y-axis). The stacked columns represent the percentages of the WT and B.1.1.7 (right Y-axis).

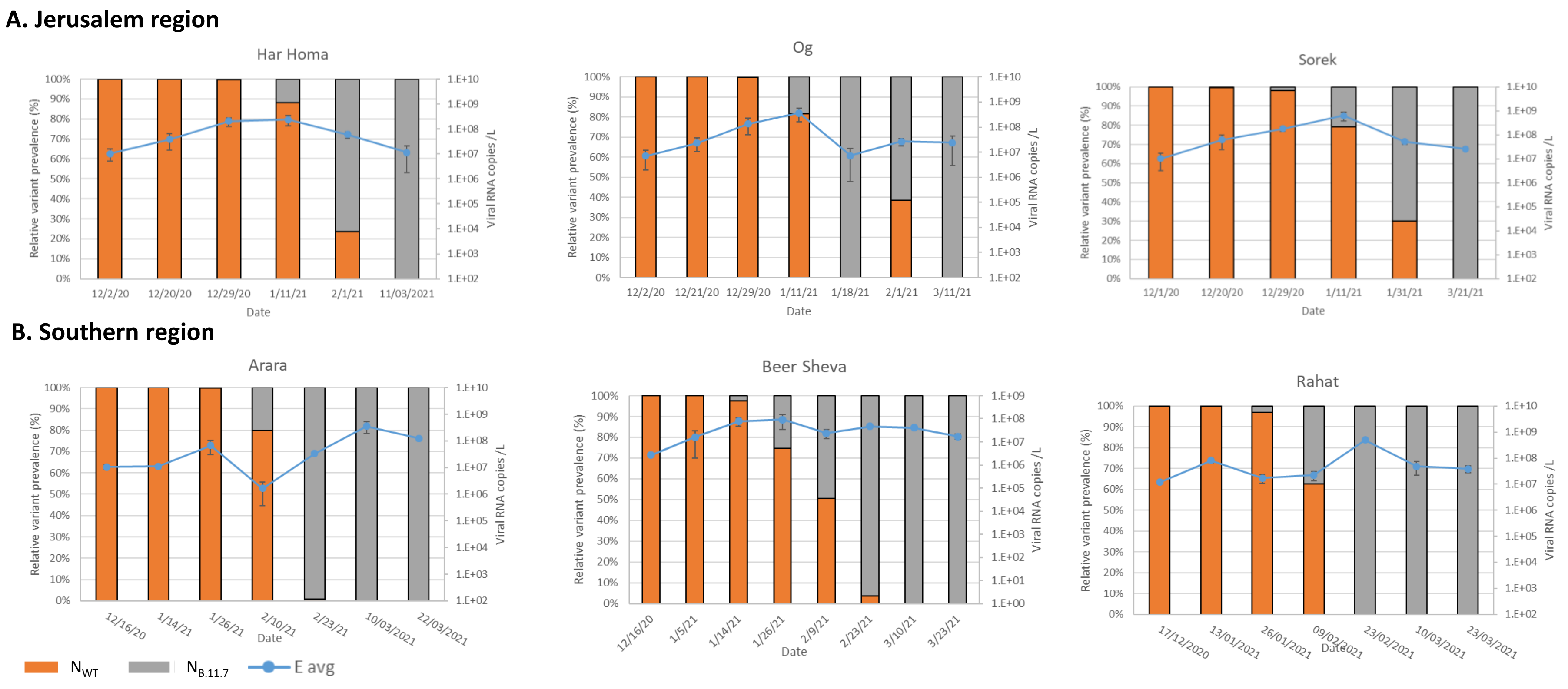

Figure S5. SC-2 total load and variant dynamics in the WWTPs of the Jerusalem district (A) and Southern (B) regions. The E gene reaction (line with markers) represent the total SC-2 viral RNA copies (left Y-axis). The stacked columns represent the percentages of the WT and B.1.1.7 (right Y-axis).

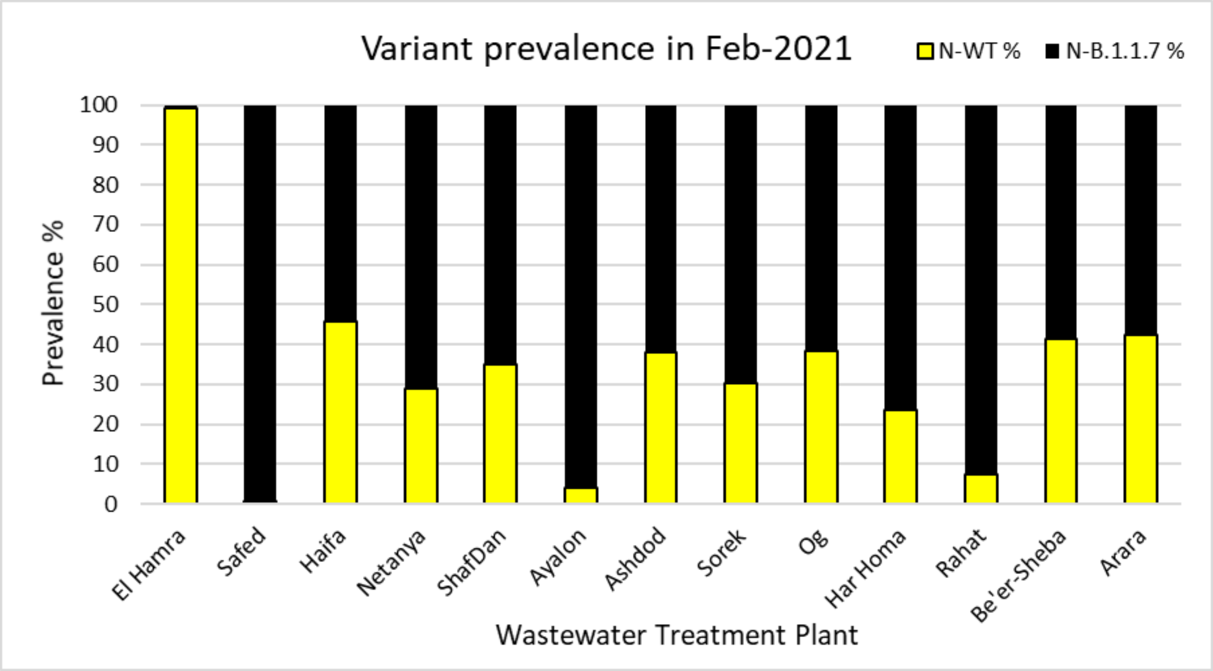

Figure S6. Average variant prevalence in each WWTP during February 2021. The average from all the measurements in each WWTP was calculated and the relative percentage of each variant.
